# Comparison of apixaban versus aspirin for the prevention of latent bioprosthetic aortic valve thrombosis: study protocol for a prospective randomized trial

**DOI:** 10.1186/s13063-024-08175-w

**Published:** 2024-05-16

**Authors:** Tomislav Kopjar, Hrvoje Gasparovic, Maja Hrabak Paar, Daniel Lovric, Petra Cerina, Tomislav Tokic, Davor Milicic

**Affiliations:** 1 Department of Cardiac Surgery, University Hospital Center Zagreb, Kispaticeva 12, 10000 Zagreb, Croatia; 2 Department of Radiology, University Hospital Center Zagreb, Zagreb, Croatia; 3 Department of Cardiovascular Diseases, University Hospital Center Zagreb, Zagreb, Croatia; 4 University of Zagreb School of Medicine, Zagreb, Croatia

**Keywords:** Aortic valve replacement, Biological valve, Valve thrombosis, Rapid deployment valve, Sutureless valve, Antithrombotic therapy, Hypo-attenuated leaflet thickening, Reduced leaflet motion

## Abstract

**Background:**

The optimal antithrombotic strategy early after aortic valve replacement surgery with a biological valve remains controversial due to lack of high-quality evidence. Either oral anticoagulants or acetylsalicylic acid should be considered for the first 3 months. Hypo-attenuated leaflet thickening on cardiac computed tomography has been associated with latent bioprosthetic valve thrombosis and may be prevented with anticoagulation. We hypothesize that anticoagulation with apixaban is superior to single antiplatelet therapy with acetylsalicylic acid in reducing hypo-attenuated leaflet thickening of bioprosthetic aortic valve prostheses.

**Methods:**

In this prospective, open-label, randomized trial, patients undergoing isolated aortic valve replacement surgery with rapid deployment bioprosthetic valves will be randomized. The treatment group will receive 5 mg of apixaban twice a day for the first 3 months and 100 mg of acetylsalicylic acid thereafter. The control group will be administered 100 mg of acetylsalicylic acid once a day, indefinitely. After the 3-month treatment period, a contrast-enhanced electrocardiogram-gated cardiac computed tomography will be performed to identify hypo-attenuated leaflet thickening of the bioprosthetic valve. The primary objective of the study is to assess the impact of apixaban on the prevention of hypo-attenuated leaflet thickening at 3 months. The secondary and exploratory endpoints will be clinical outcomes and safety profiles of the two strategies.

**Discussion:**

Antithrombotic therapy after aortic valve replacement is used to prevent valve thrombosis and systemic thromboembolism. Latent bioprosthetic valve thrombosis is a precursor of clinically significant prosthetic valve dysfunction or thromboembolic events. The hallmark feature of latent bioprosthetic valve thrombosis is hypo-attenuated leaflet thickening on cardiac computed tomography. Subclinical leaflet thrombosis occurs frequently in bioprosthetic aortic valves, more commonly in transcatheter than in surgical valves. There is no evidence on the effect of direct oral anticoagulants on the incidence of hypo-attenuated leaflet thickening after surgical aortic valve replacement with rapid deployment bioprostheses.

**Trial registration:**

ClinicalTrials.gov NCT06184113. Registered on December 28, 2023

## Introduction

### Background and rationale {6a}

With continuous aging of the population, degenerative valve disease is on the rise [1]. About 21,000 isolated aortic valve replacements (AVR) were performed in 2019 in the United States [2]. The implantation of bioprosthetic heart valves is increasing rapidly due to patient preference, aging population, and the emergence of transcatheter aortic valve implantation (TAVI). More than 85% of first-time isolated AVRs employ tissue valves in the United States [3]. Tissue valve implantation has been considered a superior option to avoid anticoagulation, although recently, a significant incidence of previously unrecognized leaflet thrombosis associated with bioprosthetic valves has been demonstrated [4, 5].

Hypo-attenuated leaflet thickening (HALT) and reduced leaflet motion (RLM) can be observed on cardiac computed tomography (CT) after bioprosthetic AVR. Reportedly, HALT is more frequent after TAVI than what has been observed after AVR. Reduced leaflet motion due to HALT has been associated with subclinical (latent) bioprosthetic valve thrombosis [5]. Overt signs of valve thrombosis appear with increased gradient and symptoms of congestion. Latent thrombosis has been reported in 12% of patients receiving oral anticoagulants and in 32% of patients on dual antiplatelet therapy after TAVI [6]. Among patients following AVR, the incidence of HALT has been reported to be between 5 and 39% at different intervals from surgery [4, 7–9]. The clinical relevance of these results has yet to be placed into perspective.

There are two approaches to antithrombotic therapy of bioprosthetic heart valves following AVR [10]. One is based on oral anticoagulant therapy with vitamin K antagonists (VKA) and the other on single antiplatelet therapy with low-dose acetylsalicylic acid (ASA). Either of these regimes are indicated for the first 3 months following implantation. However, oral anticoagulation with VKA is limited by nonadherence to prescribed medications, drug discontinuation, underdosing, and poor control of the international normalized ratio.

Studies published till date comparing different strategies of antithrombotic therapy for bioprosthetic heart valves after AVR are based on stented, conventional valves [11–13]. These valves require suturing the valve sewing ring for the aortic annulus. Newer generation, rapid deployment valves do not require conventional valve suturing. Compared to conventional valves, these valves are known for their ease of implantation and excellent hemodynamic profile. When using these valves, positioning is facilitated with a balloon expandable stent. It has been shown that rapid deployment valves are a safe and effective alternative to conventional valves for AVR and are associated with excellent clinical outcomes [14]. Thus far, there have been no studies focusing on the incidence of HALT among the new generation rapid deployment surgical valves.

Direct oral anticoagulants (DOAC) have been used for the prevention and treatment of thromboembolic events in multiple clinical scenarios. When compared to VKA, it has been shown that they reduce the risk of major, intracranial, and gastrointestinal bleeding [15]. When compared to other DOACs, apixaban has been associated with a reduced risk of major bleeding complications regardless of treatment indication [15–17]. No randomized studies reporting on the effect of apixaban and the prevalence of HALT after AVR with bioprostheses have been reported to date. Therefore, in this study, we aim to evaluate whether an apixaban-based strategy in the first 3 months following AVR with novel rapid deployment valves, compared to an antiplatelet-based strategy, is superior in reducing HALT on cardiac CT.

### Objectives {7}

#### Primary objective

The primary objective of the study is to assess the impact of apixaban-based antithrombotic therapy (intervention arm) as compared with an ASA-based single antiplatelet therapy (control arm) in the prevention of latent bioprosthetic valve thrombosis associated with rapid deployment valves after a 3-month treatment period.

#### Secondary objective

The secondary endpoints will be clinical data and safety outcomes. The safety endpoint will be the incidence and severity of bleeding across the treatment and control groups, adjudicated at 3 months postoperatively.

#### Exploratory objectives

Exploratory objectives will assess the rates of HALT, RLM, New York Heart Association (NYHA) functional class, bioprosthetic valve deterioration, components of the safety composite, and a time-to-event analysis of the safety composite. The clinical outcomes will include the incidences of stroke, myocardial infarction, need for reoperation, and mortality.

### Trial design {8}

This study is designed as a prospective, randomized, open-label, blinded endpoint, single-site superiority trial with two parallel groups. Patients will be randomized with a 1:1 allocation ratio. The trial is investigator initiated; it is planned, funded, and conducted without industry involvement.

## Methods: participants, interventions, and outcomes

### Study setting {9}

Patients with severe aortic stenosis referred to AVR surgery will be recruited at the study site. The study will take place at the University Hospital Center Zagreb where patient enrollment will take place. University Hospital Center Zagreb is the main tertiary academic hospital in Croatia with the largest cardiac surgery unit performing about 1000 open heart procedures yearly.

Aortic valve replacement surgery will be performed following our standardized institutional protocol for isolated AVR surgery. Briefly, the standard for isolated AVR at our institution is a minimally invasive approach. A partial upper j-sternotomy, central cannulation cardiopulmonary bypass, and del Nido cardioplegia are used. Rapid deployment pericardial valves, either the Perceval Plus (Corcym, Milan, Italy) or the Edwards Intuity Elite (Edwards Lifesciences Corporation, Irvine, California, USA), are going to be used based on surgeon’s discretion. Valve sizing and deployment will follow the instructions for use provided by the manufacturers. This also pertains to the pressure balloon inflation during valve deployment. Postoperatively, the patients will be admitted to a dedicated cardiac intensive care unit. On the second postoperative day patients are transferred to an intermediate care unit. Early postoperative care will focus on fluid management, electrolyte balance, atrial fibrillation prophylaxis, respiratory physiotherapy, and early mobilization. Detailed postoperative antithrombotic therapy protocol is described below.

### Eligibility criteria {10}

All patients aged 65 or older with severe aortic stenosis and no indication for anticoagulation who will undergo first-time isolated AVR at the University Hospital Center Zagreb will be screened for eligibility to participate in the study. Aortic valve replacement will be performed with new generation rapid deployment bioprosthetic aortic valves. Indications for surgical treatment of aortic valve disease will be based on Heart Team discussions, according to the guidelines and standard medical practice. Screening of subjects for inclusion in the research will be done prior to surgery after the admission to the Department for Cardiac Surgery at the University Hospital Center Zagreb.

Definition of successful AVR will be based on echocardiographic criteria and the absence of complications during hospital stay. These echocardiographic criteria should be met on the postoperative ultrasound of the heart prior to hospital discharge: mean aortic valve gradient < 20 mmHg, the highest transvalvular velocity 3.0 m/s, and absence of mild or greater aortic regurgitation. Absence of complications will include stroke, acute myocardial infarction, reoperation for bleeding or any other reason, and clinically significant valve thrombosis prior to hospital discharge.

#### Inclusion criteria

1. Men and women aged 65 or older with aortic valve stenosis undergoing successful isolated first-time AVR with a rapid deployment bioprosthetic valve
2. Signed informed consent to participate in the research

#### Exclusion criteria

1. Indications for long-term use of anticoagulant therapy
2. Indications for dual antiplatelet therapy
3. Contraindications to anticoagulation or antiplatelet therapy
4. Inability to start the study drug within the planned randomization period
5. History of atrial fibrillation or atrial fibrillation lasting longer than 48 h after surgery
6. Known hemorrhagic diathesis (thrombocytopenia ≤ 50,000/mm^3^, anemia < 85 g/L, history of intracranial bleeding, subdural hematoma, gastrointestinal bleeding, or any other non-traumatic bleeding that required replacement of blood or blood products)
7. Presence of hemodynamically significant coronary artery disease or other valvular pathologies
8. Prior open-heart surgery
9. Presence of liver failure (Child-Pugh B or C) or some other disease associated with coagulopathy
10. Aortic valve infective endocarditis
11. Severe renal failure (estimated glomerular filtration rate < 30 mL/min/1.73 m^2^) or dialysis
12. Allergy to iodine contrast or other contraindication for contrast CT

### Who will take informed consent? {26a}

Written informed consent will be obtained from participants by a trained research assistant or a surgeon at the admission to the Department of Cardiac Surgery University Hospital Center Zagreb. Those who agree to join the study will be asked to provide written consent and will be screened for eligibility. Participants will have sufficient time to ask questions. Study staff will make sure to underscore that participation is voluntary and that declining to join the study does not influence in any way the standard of care provided to patients. Points that will be addressed by the study personnel obtaining informed consent include explaining why the current research study is being conducted, the sources of funding for the project, and what purpose the proposed research serves. The importance of maintaining participant confidentiality and that withdrawing from the study is at the participant’s discretion at any time of the study duration will be emphasized.

All informed consent processes will adhere to the policies set forth by the Institutional Review Board of the University Hospital Center Zagreb. The informed consent form will also contain a section dedicated to explaining what constitutes protected health information and how this information remains confidential. Finally, the informed consent form will provide contact information for the principal investigator (PI).

### Additional consent provisions for collection and use of participant data and biological specimens {26b}

Participant data and biological specimens will not be used in ancillary studies.

## Interventions

### Explanation for the choice of comparators {6b}

Placebo was not chosen as control in this study, for two reasons: (1) single antiplatelet therapy with ASA or VKA are considered the standard of care for antithrombotic prophylaxis in the first 3 months following AVR with a bioprosthetic valve, and (2) placebo would mitigate the positive effect of antithrombotic therapy as standard clinical practice.

### Intervention description {11a}

All eligible patients must provide written, informed consent before randomization to antithrombotic treatment with either apixaban for 3 months followed by ASA indefinitely (intervention) or to ASA indefinitely (control). Randomization is performed in a 1:1 ratio.

Patients randomized to the intervention arm will receive an open-label anti-Xa type DOAC, apixaban without addition of an antiplatelet drug. The treatment will be initiated on the fifth postoperative day. Planned postoperative invasive procedures (such as pacemaker implantation) would temporarily delay commencement of apixaban, which would then be continued for 3 months. Thereafter, treatment will be converted to ASA 100 mg daily indefinitely. The dosing of apixaban follows standard clinical practice for anticoagulation. The standard dose of apixaban is 5 mg twice daily. The dose should be reduced to 2.5 mg twice daily if 2 or more of the following criteria are present: (1) age ≥ 80 years, (2) body weight ≤ 60 kg, (3) serum creatinine ≥ 1.5 mg/dL (133 μmol/L).

Patients assigned to the control arm will receive an open-label ASA 100 mg per day. The treatment will be initiated on the fifth postoperative day and continued indefinitely. The current standard dosing of ASA is 75–100 mg daily. There are no additional dose adjustments for ASA. In patients who are intolerant to ASA, conversion to 75 mg of clopidogrel a day will be recommended. Medication compliance will be self-reported by trial participants, resembling clinical practice.

Antithrombotic therapy based on the study protocol will start at the time of randomization which will be on the fifth postoperative day. Patients participating in the study should take the study drug according to the study protocol every day for a period of 90 days. Prior to randomization, starting from the first postoperative day, antithrombotic therapy with low molecular weight heparin (e.g., enoxaparin, dalteparin, or fondaparinux) will be employed without addition of antiplatelet or oral anticoagulant medications.

### Criteria for discontinuing or modifying allocated interventions {11b}

Patients randomized to the control arm who develop an indication for anticoagulation (e.g., atrial fibrillation) during follow-up will cross over to the apixaban group. Conversely, patients in the intervention group who develop an indication for antiplatelet therapy (e.g., percutaneous coronary intervention) will cross over to the ASA group. All patients will remain as originally allocated for the intention-to-treat analysis of initial strategy. Per-protocol analysis will be performed for safety composite outcomes.

An investigator may withdraw a participant from the study for the following reasons: (1) significant study intervention non-compliance; (2) identification of a clinical adverse event, laboratory abnormality, or other medical condition which could harm the patient in any way by continued participation in the study; (3) if the participant meets an exclusion criterion that precludes further participation; (4) participant requests withdrawal from the trial.

In case of an overt valve thrombosis, ischemic stroke, or systemic embolism before the end of the study, patients will be managed according to the best standards of care regardless of the study protocol allocation. Additional cardiac imaging including a heart ultrasound (transthoracic and/or transesophageal) and, if necessary, a cardiac CT will be considered. The result of this additional imaging as well as previous imaging exams of the heart will be available to the treating physician for evaluation and decision on the appropriate antithrombotic treatment. If necessary, the study drug will be stopped and anticoagulant therapy with unfractionated heparin and/or a VKA will be introduced. On the other hand, if an overt Valve Academic Research Consortium 3 (VARC-3) Type 2–4 bleeding event [18] occurs, the study drug will be discontinued. In any case, immediate medical care, and treatment, including hospitalization will be available.

### Strategies to improve adherence to interventions {11c}

Adherence to protocol will be indicated by attending all the scheduled visits, conducting the telephone interviews, and undergoing planned echocardiographic and cardiac CT imaging as well as taking at least 95% of their medication. Several consecutive attempts during the week of the scheduled visit will be made to reschedule a participant’s visit. Medication compliance will be evaluated by counting returned tablets; participants will be asked to bring their medication at every visit. Participants will also be asked if there was any difficulty in adhering to the medication taking during each visit or telephone interview.

### Relevant concomitant care permitted or prohibited during the trial {11d}

The study will include men and women aged 65 and older. It is to be expected that the patients participating in the study should receive concomitant pharmacotherapy for their comorbid conditions and age-related diseases. Common conditions for this age group are cardiovascular diseases, hypertension, diabetes mellitus, and others. Participants receiving concomitant pharmacotherapy will be encouraged to continue with medical treatment of their diseases. This is to ensure that the patients will receive the appropriate medical care which will not interfere or confound the effect of the study drug on outcome measures.

### Provisions for post-trial care {30}

If a participant experiences any harm from known or unknown risks of the research procedures as described, immediate medical care and treatment, including hospitalization, if necessary, will be available. There is currently no consensus on the treatment of isolated HALT without clinical symptoms. If HALT of the bioprosthetic valve is established on cardiac CT at the end of the study, and the patient experiences symptoms judged to be related to valve degeneration or is found to have stage 2 or 3 valve deterioration on echocardiogram [18], treatment will be considered. Trial-specific investigations will be made available to independent clinicians who will evaluate whether pharmacological or interventional therapy is indicated according to best clinical practice. Immediate medical care including hospitalization, if necessary, will be available.

### Outcomes {12}

#### Primary outcome measure

**Fig. 1:**
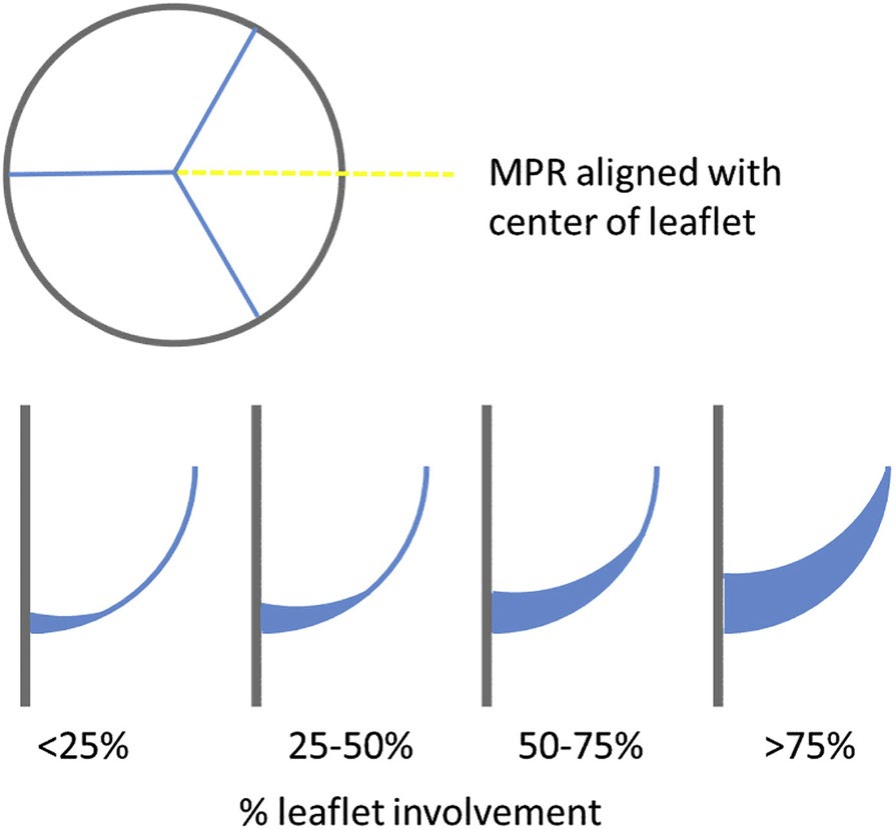
Multiplanar reformation alignment and semi-quantitative grading of attenuated leaflet thickening. Reprinted with permission from Blanke et al. [19]. MPR, multiplanar reformation

The primary outcome of the study is to assess whether an anticoagulation-based strategy with apixaban during the first 3 months after rapid deployment bioprosthetic AVR can reduce the prevalence of HALT when compared to single antiplatelet-based strategy with ASA. A cardiac CT will be performed to assess for the presence or absence of HALT associated with the bioprosthetic valve. Hypo-attenuated leaflet thickening is typically identified as increased thickness of one or more leaflets of the bioprosthetic valve on contrast-enhanced electrocardiogram (ECG)-gated cardiac CT in at least two reconstructed planes using 2D multiplanar reformatted images. A meniscal-shaped configuration is exhibited, starting thickest at the insertion of the bioprosthetic leaflet and gradually tapering towards the leaflet edge (Fig. 1) [19]. The extent of HALT should be described using a 4-tier semiquantitative grading scale regarding leaflet involvement along the curvilinear contour in diastole: (1) ≤ 25%, (2) > 25% and ≤ 50%, (3) > 50% and ≤ 75%, (4) > 75% [20]. The definition of HALT for the purposes of this study is going to be based on those bioprosthetic valves where more than 50% of at least one valve leaflet involvement can be described. This translates to a grade 3 or higher on a semiquantitative 4-tier grading scale. Proportions of HALT will be compared between the study groups. This outcome will be assessed for superiority of apixaban versus ASA. All cardiac CT examinations will be blindly adjudicated at a core lab.

#### Secondary outcome measures

The secondary outcome will assess whether the treatment with apixaban could lead to more bleeding events or whether withholding ASA could lead to more thromboembolic events. This will be assessed through a safety composite outcome measure. The safety composite outcome will be comprised of all types of VARC-3 bleeding events, thromboembolic events (myocardial infarction and stroke), and death from any cause, at 3 months after randomization. A descriptive classification scheme for VARC-3 bleeding, like the Bleeding Academic Research Consortium classification has been proposed. It includes 4-type bleeding scale: type 1 (minor), type 2 (major), type 3 (life-threatening), and type 4 (leading to death) bleeding [18]. This outcome will be assessed for noninferiority of apixaban versus ASA at 3 months after randomization.

#### Exploratory outcome measures

Exploratory outcome measures will include the rates of HALT, RLM, NYHA functional classes, bioprosthetic valve deterioration, VARC-3 bleeding events (types 1–4), myocardial infarction, stroke, need for reoperation, and all-cause mortality. Time-to-event analysis of the safety composite will be employed as well.

Reduced leaflet motion in the presence of HALT can be identified on cardiac CT or transesophageal echocardiography. The strength of cardiac CT is its high spatial resolution. Leaflet restriction caused by HALT can be described as RLM, although it may or may not be present with HALT. Assessing leaflet motion in the absence of HALT increases the likelihood of false positive diagnosis of RLM. Using CT, RLM will be assessed on short-axis reformatted images during systole and 4D volume rendering images. Once HALT has been confirmed the extent of RLM can be described per leaflet, using a 4-tier grading scale in systole: (1) not present, (2) < 50% restriction in leaflet excursion, (3) ≥ 50% restriction in leaflet excursion, and (4) immobile leaflet [20].

Bioprosthetic valve deterioration is a progressive process that requires serial longitudinal assessments of clinical status as well as valve morphology, function, and hemodynamics. Stages of bioprosthetic valve deterioration have been proposed though hemodynamic criteria assessed by echocardiography as suggested in VARC-3 [18]. Briefly, stage 1 is described with morphological valve deterioration without significant hemodynamic changes. Stage 2 is characterized with moderate hemodynamic valve deterioration with an increase in mean transvalvular gradient ≥ 10 mmHg, and stage 3 with severe hemodynamic valve deterioration with an increase in mean transvalvular gradient ≥ 20 mmHg.

### Participant timeline {13}

**Fig. 2:**
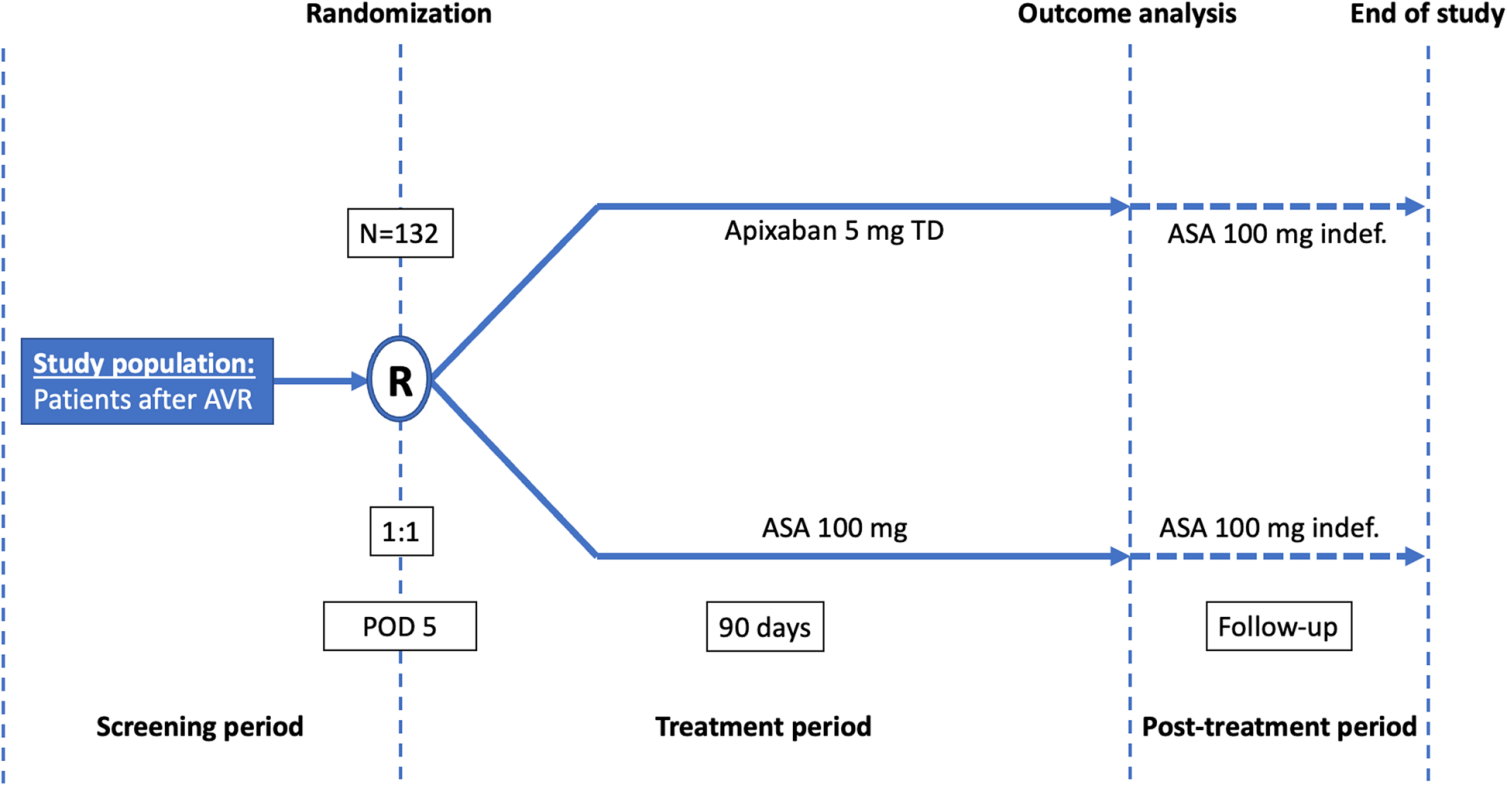
Study schematic diagram. ASA, acetylsalicylic acid; AVR, aortic valve replacement; POD, postoperative day; R, randomization; TD, twice daily

A schematic diagram of the study is presented in Fig. 2. The total study duration for participants will be up to 3 months. During the 90-day study period, patients will be taking their assigned medication and attending one clinical visit 6 weeks after surgery outside the study protocol. They will undergo two monthly telephone interviews. At the end of the 90-day treatment period, they will be invited for a clinical visit when they will undergo a planned cardiac CT and heart ultrasound.

**Fig. 3:**
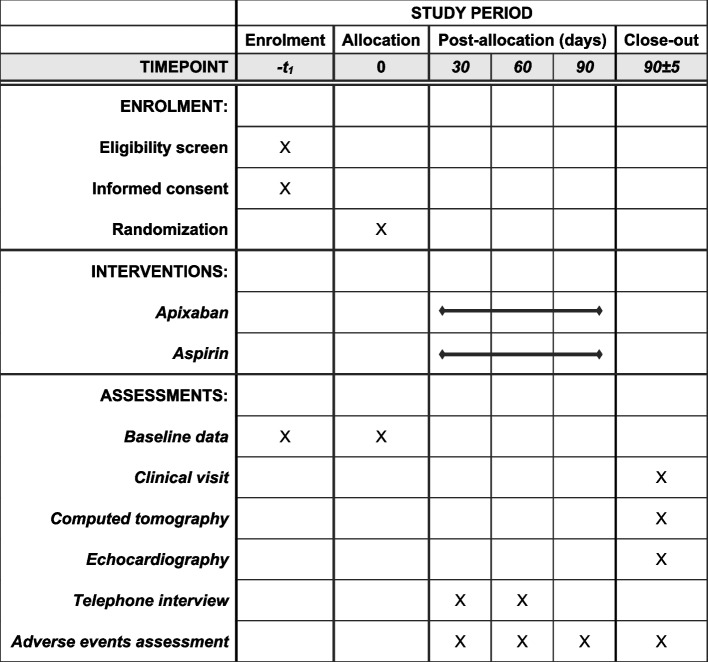
Standard Protocol Items: Recommendations for Interventional Trials schedule of enrolment, interventions, and assessments

**Fig. 4:**
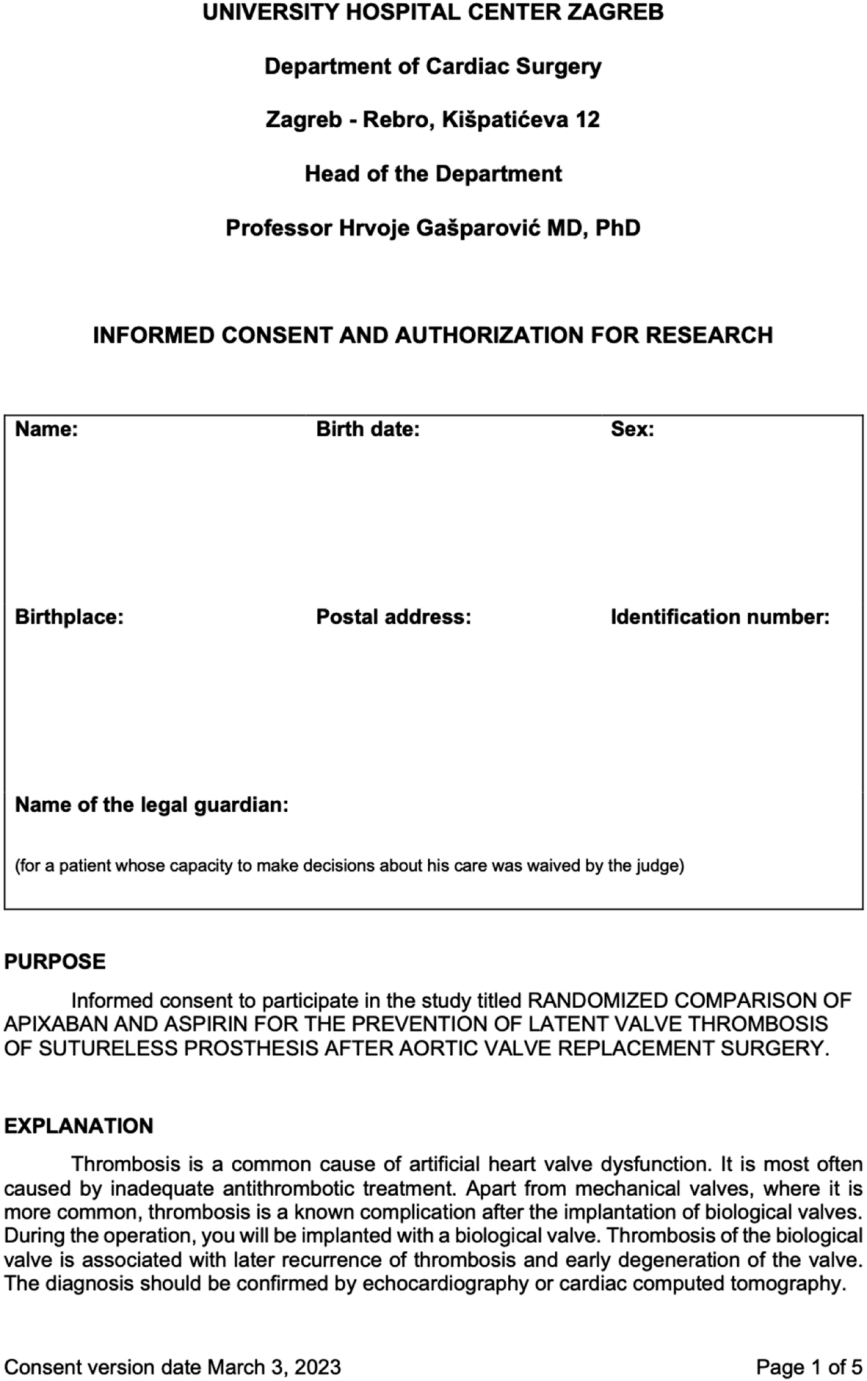
Consent form

A timeline of enrolment, interventions, and assessments is provided in Fig. 3. Participants will initially be screened upon admission to the Department of Cardiac Surgery at the University Hospital Center Zagreb. During screening, all the study inclusion and exclusion criteria will be analyzed, particularly any potential indication for long-term oral anticoagulation or dual antiplatelet therapy or history of hemorrhagic diathesis. If deemed eligible and after signing the informed consent (Fig. 4), they will be scheduled for randomization on the fifth postoperative day following first time isolated rapid deployment AVR surgery. Prior to hospital discharge patients will undergo a planned hemodynamic evaluation of the newly implanted rapid deployment bioprosthetic aortic valve with echocardiography to confirm the definition of successful AVR. Absence of complications will include stroke, acute myocardial infarction, reoperation for bleeding or any other reason, and clinically significant valve thrombosis prior to hospital discharge.

After hospital discharge, monthly telephone interviews will be conducted to remind the patients of their planned visit and to get information on any complications or unexpected events that might have occurred in the meantime. They will be asked about any side effects, and the number of remaining tablets to determine treatment adherence. Participants’ clinic visit based on the study protocol will take place 90 ± 5 days from the time of randomization. They will be interviewed for clinical and adverse events once again. Side effects, adverse events, any unplanned hospital or emergency room visits, or any bleeding events will be noted. The number of remaining tablets will be counted to determine treatment adherence. A scheduled cardiac CT and heart ultrasound will be performed.

### Sample size {14}

According to previous studies tracking HALT of bioprosthetic heart valves, the rate has been estimated to be up to 39% [4, 7–9] among surgical valves. It has been estimated that there might be a 60–70% decrease in the incidence of HALT of bioprosthetic valves among patients receiving oral anticoagulants when compared to antiplatelet therapy [21]. If it is to be expected that HALT of the biological valve in the study will occur in about 30% of control patients, a premise of a 60% decrease in the prevalence after a 3-month treatment period seems reasonable and relevant. The null hypothesis is that compared to the ASA controls, apixaban treatment will not mitigate HALT prevalence among patients after rapid deployment AVR following a 3-month treatment period. With 158 participants randomized 1:1, we expect to have 80% power (type I error < 0.2) to detect a significant difference (type I error < 0.05) between the study groups, thus rejecting the null hypothesis. To account for protocol violation, loss to follow-up, and withdrawal of consent in 5% of participants, we aim to randomize at least 166 patients.

### Recruitment {15}

The accrual rate is expected to be up to 7 participants per month with the recruitment period spanning over 24 months. We predict the need to screen about 200 individuals to meet the goal of enrolling 166 study participants. Recruitment sources include the Department of Cardiac Surgery University Hospital Center Zagreb. All potential participants will be logged in a password protected database on a secured, shared server, which will contain their contact information, date of contact, eligibility, recruitment source, and date of scheduled visit; identifiable health information will not be stored during recruitment.

## Assignment of interventions: allocation

### Sequence generation {16a}

We will use the R software to generate a random sequential list of binary codes by participant number. Blocks of fixed length will be applied to keep the number of both arms relatively balanced. Set seed function will be used to ensure that the randomization schedule is reproducible. Participants who have been screened and meet criteria will enter the randomization section and be assigned with the appropriate number and corresponding intervention upon confirmation at the first clinic visit.

### Concealment mechanism {16b}

Only the (PI) will know what code corresponds to which treatment arm. Trial randomization codes will be kept on a password-protected Excel sheet located on a secure server to which only the PI will have access to.

### Implementation {16c}

Trained research assistants will enroll participants. Once a patient has been successfully screened, a binary code that is to be allocated to each participant will be generated. Participants will be assigned to interventions based on whichever binary code they are allocated.

## Assignment of interventions: blinding

### Who will be blinded {17a}

Since this is a prospective, randomized, open-label, blinded endpoint trial all outcomes will be adjudicated in a blinded fashion to preserve impartiality. All operators performing CT or echocardiographic analysis will be blinded to study group allocation at the time of image acquisition. Imaging will be assessed off-line in a blinded manner. This level of blinding is maintained throughout the conduct of the trial, and only when the data has been analyzed according to statistical analysis plan, and conclusions regarding the primary and secondary outcomes are made, will the associated personnel be unblinded. Even though the adjudication of clinical events in the safety composite is blinded, we cannot exclude that bias may be introduced by the trial staff when filling the case report files. Data monitoring will be performed to minimize this.

### Procedure for unblinding if needed {17b}

Not applicable since this is an open-label trial.

## Data collection and management

### Plans for assessment and collection of outcomes {18a}

After signing the informed consent and inclusion into the study, patients’ baseline data will be collected, including medical history and concomitant medication. Data from echocardiograms obtained before and after AVR will be collected. Data on adverse clinical events and study protocol adherence during the treatment period will be collected through telephone interviews at 1 and 2 months following randomization. Three months following randomization, all study outcomes will be assessed. Patients will be invited for an on-site clinical visit. The visit will include an extensive interview to assess for any potential clinical, adverse event, hospital or emergency room visits, or bleeding events that might have occurred during the study period. Reports from any hospital or emergency room visits will be obtained and analyzed. During the same visit a cardiac CT and echocardiography will be performed (Fig. 3).

#### Computed tomography

Computed tomography examination will be performed 90 ± 5 days from the moment of randomization on a 128-layer CT device with intravenous administration of iodine contrast medium and synchronization of the recording with the ECG. The previously described CT acquisition protocol will be employed [20]. Briefly, image contrast medium, 80–100 mL, will be administered intravenously through an intravenous cannula at least 20 gauge wide at a rate of 4–6 mL/s. For the optimal representation of the leaflets, imaging will be performed throughout the entire cardiac cycle without radiation dose modulation, with image reconstruction every 5% R-R interval (20 separate phases) with a slice thickness of 0.6 mm. In most patients, an X-ray tube voltage of 100–120 kV will be used, exceptionally 140 kV for extremely obese patients or patients with pacemakers. To achieve an optimal pulse rate during imaging (< 70/min.), if necessary, patients will be premedicated with beta-blockers and/or ivabradine. Imaging will begin when the contrast agent density in the ascending aorta reaches 300 Hounsfield units, and the imaging field will be limited to the heart area.

Hypo-attenuated leaflet thickening is identified by a contrast CT of the heart as an increased thickness of the bioprosthetic leaflet. A typical thickening assumes a meniscus-shaped configuration. The thickening begins and is thickest at the point of insertion of the leaflet for the valve frame and gradually narrows towards the free edge of the leaflet. The extent of HALT is assessed by a semiquantitative scale for each leaflet separately. The percentage of leaflet involvement is estimated starting from the leaflet base. The assessment is performed using multiplanar reconstructions.

The advantage of CT is its high spatial resolution. Sometimes, the assessment of HALT can be limited by artifacts caused by the valve stent frame, motion artifact, or suboptimal contrast, making the computed tomography study insufficient for diagnosis. Leaflet restriction caused by HALT can be described as RLM. However, due to the limited temporal resolution of CT of the heart, RLM should be investigated only in cases of a diagnosis of HALT. Otherwise, the probability of a false positive diagnosis increases. In the literature, HALT and RLM are used mainly as synonyms for subclinical (latent) leaflet thrombosis of bioprosthetic heart valves. The extent of HALT will be described per leaflet, using a 4-tier semiquantitative grading scale as described previously [20]. Grade 3 or higher will be considered HALT positive valve for the purpose of this study. Similarly, in the presence of HALT, the extent of RLM will be assessed per leaflet on multiplanar reformatted and 4D volume rendered images, using a 4-tier semiquantitative grading [20].

A specialist radiologist will evaluate the degree of HALT and RLM by analyzing a contrast enhanced CT study of the heart. The radiologist will be blinded to the patient’s pharmacotherapy or to which group the patient has been randomized.

#### Echocardiography

Echocardiography is a widely accessible and validated method of assessing bioprosthetic valve function. An echocardiographic examination will be performed on two occasions during the study. The first one will be done during the index hospitalization, after AVR prior to discharge. It will assess for the definition of successful aortic valve replacement. The second echocardiographic examination will be done 90 ± 5 days after randomization. Repeated echocardiograms at baseline and at 3 months will determine the degree of hemodynamic valve deterioration as suggested in VARC-3 [18]. All echocardiograms will be blindly adjudicated at a core lab.

Echocardiographic examinations will be based on transthoracic echocardiography (TTE) and guidelines for echocardiographic assessment of prosthetic valves [22]. Transthoracic echocardiography is the gold standard in assessing the hemodynamic characteristics of bioprosthetic heart valves. It follows the same principles as for native heart valves although it has some peculiarities. Two-dimensional TTE will be used as the first line of imaging, and three-dimensional transesophageal echocardiography (TEE) will be performed in cases of bioprosthetic dysfunction. Comprehensive echocardiographic imaging of a prosthetic valve involves the use of multiplanar views with care to confirm appropriate leaflet morphology and motion and to identify the presence of calcifications or structural abnormalities of any component of the prosthesis.

Transthoracic echocardiography Doppler imaging will be performed at an imaging speed of 100 mm/s. The parameters that will be recorded are the bioprosthetic aortic valve mean gradient and the effective orifice area (EOA). Measurements will be performed during two cardiac cycles in sinus rhythm. In the case of atrial fibrillation, Doppler measurements will be performed during the period of physiological heart rate and through five cycles. For EOA calculations, the continuity equation obtained from the stroke volume will be used. It is recommended to adjust the cycle length to 10%. Doppler imaging will be done during quiet breathing or in the middle of expiratory apnea.

Color Doppler will show the presence of pathological intra- and/or paravalvular regurgitation blood flows, if any. The search will be performed in multiple sequences with appropriate setting (Nyquist limit around 50–60 cm/s). Accurate localization of paravalvular regurgitation can be challenging. In this case, TEE is necessary to assess the localization and degree of regurgitation.

### Plans to promote participant retention and complete follow-up {18b}

Participants will be provided the medication, clinic visit, echocardiography, and CT at no additional costs. Participants will be thoroughly educated about the risks and benefits of the study drugs. All medical procedures will be performed by trained clinical staff. Participants will be encouraged to communicate with research staff via telephone regarding any problems or irregularities. Adherence to protocol involves attending the clinical visit and taking at least 90% of their medication. During the week of the scheduled visit, a “no-show” will lead to three attempts to reschedule a participant’s visit. Medication compliance will be evaluated by counting returned tablets.

### Data management {19}

The data collection plan for this study is to utilize a database to capture all treatment and adverse event data for all enrolled subjects.

### Confidentiality {27}

Identifying information of the participating patients will be removed and substituted with a study participant number. All data will be de-identified. Nonetheless, we will maintain a file that links subject name with the study participant number thereby enabling us to locate the study participant’s research record if needed. Data will be analyzed in aggregate only, and no identities will be revealed. It will be specifically used for the purposes outlined in this proposal and not for any other purpose. Study documentation will be stored in a secure location until the end of the study and all data analyses are complete. At that time, all study material will be placed in a secured long-term storage facility until it is deemed appropriate to destroy the study material.

### Plans for collection, laboratory evaluation, and storage of biological specimens for genetic or molecular analysis in this trial/future use {33}

Not applicable. No genetic or molecular analysis were planned in the study protocol.

## Statistical methods

### Statistical methods for primary and secondary outcomes {20a}

The primary outcome will be tested using the chi-square test of proportions. It will be tested for superiority using a 2-sided chi-square test at the 0.05 significance level in the intention-to-treat population. The secondary outcome will be test for non-inferiority with a single comparison between the study groups of the secondary outcome measure (safety composite) per-protocol. To test for noninferiority, we determined whether the upper boundary of the 95% confidence interval for the difference in the rate of secondary outcome measure between the treatment group and the control group was less than or equal to the prespecified noninferiority margin of 5 percentage points. If this requirement for noninferiority will be met, testing for the superiority of the safety composite will be performed at a two-sided alpha level of 0.05. The Kolmogorov-Smirnov test will be used to test normality of distribution for continuous data. For exploratory outcome measures, between-group differences will be analyzed using independent samples *t*-tests, chi-square test, or Mann-Whitney tests, where appropriate. If necessary, multivariable regression analysis will be used to adjust for imbalances in main prognostic variables between the intervention and control groups. Time-to-event analysis for the safety composite outcome and its components will be performed. Cumulative event-free survival will be estimated by Kaplan-Meier analysis.

### Interim analyses {21b}

The data monitoring committee will act in an advisory role to ensure safety by reviewing the cumulative data from the clinical trial. An interim analysis will be performed once 50% of the sample has been achieved. The effects of the study will be assessed on clinical outcomes. The sample size of the study will increase, if necessary, based on the interim analysis. The monitoring committee may recommend modifying or terminating a clinical trial based on any perceived safety concerns, regardless of statistical significance. The sponsor is authorized to stop the study at any time if the safety of the participants is compromised.

### Methods for additional analyses (e.g., subgroup analyses) {20b}

Not applicable. No plans for additional analyses.

### Methods in analysis to handle protocol non-adherence and any statistical methods to handle missing data {20c}

Study population will be randomized to the intervention (apixaban) or the control (ASA) arm. Carefully selected patients based on the inclusion/exclusion criteria will provide a basis for a steady protocol adherence. In 5% of patients, a protocol violation, loss to follow-up, and withdrawal of consent are anticipated. All patients will remain in their originally allocated arm for the intention-to-treat analysis of all outcomes. Exploratory analyses will include a per-protocol analysis, which will include only patients who received their randomly assigned treatment, and an as-treated analysis in which we will compare patients who received apixaban with those who received aspirin with multivariable adjustment for imbalances in baseline characteristics. Maximal efforts to reduce the missing data to a minimum will be employed. Missing data will be handled via multiple imputations in statistical analyses.

### Plans to give access to the full protocol, participant-level data, and statistical code {31c}

The datasets analyzed during the current study are available from the corresponding author upon a valid request.

## Oversight and monitoring

### Composition of the coordinating center and trial steering committee {5d}

The overall responsibility for the study and its management will be with the PI. The trial steering committee is comprised of senior members of the Department of Cardiac Surgery at the University Hospital Center Zagreb. The steering committee will be responsible for the day-to-day running of the trial.

### Composition of the data monitoring committee, its role and reporting structure {21a}

An independent data safety monitoring board, consisting of a specialist cardiac surgeon and an independent trial statistician, is responsible for monitoring safety during the trial. An independent trial statistician will perform the statistical analyses. No formal stopping criteria are defined; the data safety monitoring board will give recommendations of stopping the trial if they consider interim data to be convincing.

### Adverse event reporting and harms {22}

During the entire study adverse event and any unintended effect of trial interventions will be collected. Participants will be asked to report any adverse event that they might have experienced after starting the study drug. They will also be able to contact the PI via the phone number listed in the informed consent form at any time to report any clinical or adverse event. Adverse events that may cause the subject to terminate protocol treatment are bleeding events. Thromboembolic events or bioprosthetic valve dysfunction might lead to crossover. We have clearly described adverse events we are planning to collect for outcome analysis. They will be labeled in primary or secondary outcomes. These outcomes will be reported in case report forms and do not need to be reported separately.

### Frequency and plans for auditing trial conduct {23}

Although this is a low-risk intervention trial, the trial steering committee and the independent safety monitoring board will meet 4 times a year to review the conduct throughout the trial period. The Institutional Review Board has the right to audit studies.

### Plans for communicating important protocol amendments to relevant parties (e.g., trial participants, ethical committees) {25}

Protocol amendments will be submitted to the appropriate ethics committee for approval before implementation. All agreed protocol amendments will be clearly recorded on a protocol amendment form and will be signed and dated by the original protocol approving signatories. As soon as approved, the PI will notify the trial participants and the sponsor. A copy of the revised protocol will be archived in file. Any deviations from the protocol will be fully documented using a breach report form. In case of protocol amendments, they will be updated in the clinical trial registry as well.

### Dissemination plans {31a}

De-identified patient data can be made available after the study-specific aims have been published. The statistical analyses will be available for those who request it based on published analyses. Authorship of the final report will be based on contribution to the trial as determined by the PI. The final report will be published in a peer-reviewed journal. Should the study participants indicate an interest in receiving these results, they will be shared with them.

## Discussion

Valve thrombosis is a common cause of prosthetic valve dysfunction. It may be related to inadequate antithrombotic therapy. Bioprosthetic valve thrombosis is a major cause of either acute or indolent bioprosthetic valve degeneration and often has an elusive presentation causing delayed recognition and treatment.

Subclinical leaflet thrombosis is found with significant frequency among bioprosthetic valves. It is characterized by HALT and RLM on high-resolution 4-dimensional cardiac CT. While the occurrence of HALT has been described across several transcatheter and surgical bioprostheses [5, 21], its effect on long-term valve function remains unclear [4, 7]. One of the reasons for the unclear relationship between HALT and long-term valve function might be related to the natural history of subclinical leaflet thrombosis. It is possible that HALT may exhibit spontaneous progression or even regression as part of its natural history, regardless of pharmacotherapy [23]. Whether more aggressive antithrombotic strategies could mitigate the occurrence of subclinical leaflet thrombosis is a matter of ongoing debate. Recent guidelines recommend a selective use of oral anticoagulants in patients with confirmed HALT and RLM with elevated gradients (Class of recommendation IIa, Level of evidence B) [10].

Over the last two decades, minimally invasive cardiac surgery has been developing rapidly [24]. Alongside the development of minimally invasive cardiac surgery, new sutureless and rapid deployment bioprosthetic valves were developed. They were developed to reduce ischemic times by avoiding the use of sutures to fix the valve to the annulus because of a new stent configuration, which can expand and thus anchor the valve in the right position [25]. These valves are conceptually different from the conventional bioprosthetic valves that require suture anchoring for the aortic annulus. It has been suggested that the stent frame and leaflets design of rapid deployment and sutureless valves may have a potential influence of thrombus formation and, hence, risk of stroke [9]. Current knowledge about antithrombotic treatment of patients implanted with this novel biological prosthesis is very limited and is not based on randomized studies. Moreover, antithrombotic recommendations in the guidelines are lacking as well.

This is the first trial of its kind to investigate the efficacy of apixaban in the prevention of latent bioprosthetic valve thrombosis after AVR with new generation rapid deployment bioprostheses. The proposed study addresses an unanswered question regarding the prevention of silent valve thrombosis after bioprosthetic valve implantation. Direct oral anticoagulants have not been studied for antithrombotic treatment in the early postoperative period.

There are two statistical concerns with the trial. First, this is a single-center study that may limit its external validity, the results may not be generalizable to other populations or settings, and there may be variations in practice and patient characteristics that could affect the outcomes. Second, this study has limited statistical power. To overcome these issues, we will employ efforts to expand the study to multiple regional centers. Apixaban has the theoretical potential to improve subclinical biological valve thrombosis and maintain low risk of bleeding. If shown to be effective, this trial may serve to change the way of antithrombotic management after AVR, paving the way for further research.

## Trial status

This manuscript was based on protocol version 1.2, dated March 3, 2023. Recruitment for this study began in December 2023 and it is expected to be completed by the end of 2025.

## Data Availability

Any data required to support the protocol will be supplied upon reasonable request if all members of the investigative team approve the request.

## Abbreviations

ASA: Acetylsalicylic acid
AVR: Aortic valve replacement
CT: Computed tomography
HALT: Hypo-attenuated leaflet thickening
DOAC: Direct oral anticoagulant
EOA: Effective orifice area
NYHA: New York Heart Association
PI: Principal investigator
RLM: Reduced leaflet motion
TAVI: Transcatheter aortic valve implantation
TEE: Transesophageal echocardiography
TTE: Transthoracic echocardiography
VARC: Valve Academic Research Consortium
VKA: Vitamin K antagonists
